# A Case of Invasive Brevibacterium Bacteremia in a Severely Immunocompromised Patient

**DOI:** 10.7759/cureus.112013

**Published:** 2026-07-03

**Authors:** Anzi Salim, Andrew Maza, Maheswara Reddy Koppula

**Affiliations:** 1 Internal Medicine, St. Michael Medical Center, Silverdale, USA; 2 Physical Medicine and Rehabilitation, Northwell Health, New Hyde Park, USA; 3 Internal Medicine, Drexel University College of Medicine, Philadelphia, USA

**Keywords:** bacteremia, brevibacterium, immunocompromised host, opportunistic infection, vancomycin

## Abstract

*Brevibacterium* spp., previously considered nonpathogenic skin commensals, have emerged as opportunistic pathogens in immunocompromised hosts with central venous catheters or malignancies. We report a case of *Brevibacterium* bacteremia in a 47-year-old HIV-positive woman with profound immunosuppression (CD4+ T-cell count of 1 cell/µL) but notably without any indwelling catheter or prosthetic device. The patient presented with syncope and pancytopenia and was ultimately diagnosed with *Brevibacterium* bacteremia after extensive workup. She was successfully treated with a two-week course of intravenous vancomycin, with resolution of bacteremia. This case highlights the need to consider these organisms in severely immunocompromised patients, even in the absence of indwelling catheters. Vancomycin remains an effective treatment.

## Introduction

*Brevibacterium* is a genus comprising over 50 species of non-motile, catalase-positive, aerobic, Gram-positive bacilli [1]. Due to its morphological similarity to other genera, *Brevibacterium* has been difficult to classify and has been redefined several times since being first identified in 1953 [2]. Until 1969, when an infant was diagnosed with *Brevibacterium* meningitis following the placement of a ventriculocardiac shunt, *Brevibacterium* spp. were considered nonpathogenic [3]. Since then, additional patient cases have been reported, often with *Brevibacterium* bacteremia or endocarditis. Typically, these patients are immunocompromised, and the number of reported cases has increased over the last 25 years [3]. The largest risk factors include central catheter placement and immunocompromising conditions or therapies, including malignancy, chemotherapy, stem cell transplantation, and solid organ transplantation [3,4]. *Brevibacterium* spp. have been identified on human skin, genital hair, and in otorrhea. Environmental and industrial sources include dairy products such as aged cheeses, as well as ectoine, a bacterial-derived osmoprotectant used in select biomedical therapies, including ophthalmic and nasal treatments [5].

## Case presentation

A 47-year-old woman with a known medical history of cerebral palsy, HIV infection with a history of antiretroviral therapy (ART) nonadherence, hypertension, chronic obstructive pulmonary disease not requiring home oxygen, asthma, migraines, and a recent small lacunar stroke presented to the emergency department following a syncopal episode. Before the event, she experienced a prodrome consisting of nausea, diaphoresis, dizziness, and a sensation of warmth before the syncopal episode. She also reported increasing difficulty with ambulation in the weeks preceding admission.

Initial evaluation in the emergency department revealed marked leukopenia, with a white blood cell count of 1,600 cells/µL (reference range: 4,000-10,000 cells/µL). Red blood cell and platelet counts were also decreased at 3,050,000 cells/µL (4,200,000-5,400,000 cells/µL) and 102,000 cells/µL (150,000-450,000 cells/µL), respectively, indicating pancytopenia, which was considered most consistent with HIV-associated bone marrow suppression in the setting of advanced, untreated disease.

A comprehensive metabolic panel demonstrated mild elevations in aspartate aminotransferase (AST) at 79 U/L (10-40 U/L), alanine aminotransferase (ALT) at 144 U/L (7-56 U/L), and alkaline phosphatase (ALP) at 140 U/L (44-147 U/L), while amylase and lipase levels were within normal limits (Table 1). The patient was not taking any medications known to cause leukopenia or pancytopenia. She met the diagnostic criteria for systemic inflammatory response syndrome, with hypotension (blood pressure, 88/45 mmHg), tachycardia (heart rate, 91 beats/min), and significant leukopenia. Sepsis was diagnosed based on evidence of hemodynamic instability and leukopenia. Physical examination revealed oral thrush but was otherwise unremarkable. Orthostatic vital signs were negative.

**Table 1:** Laboratory findings AST: aspartate aminotransferase, ALT: alanine aminotransferase, ALP: alkaline phosphatase, CD4: cluster of differentiation 4, ↓: below the normal range, ↑: increased or above the normal range

Parameter	Patient value	Reference range	Interpretation
White blood cell count	1,600 cells/µL	4,000-10,000 cells/µL	↓ Leukopenia
Red blood cell count	3,050,000 cells/µL	4,200,000-5,400,000 cells/µL (female)	↓ Anemia
Platelet count	102,000 cells/µL	150,000-450,000 cells/µL	↓ Thrombocytopenia
AST	79 U/L	10-40 U/L	↑ Elevated
ALT	144 U/L	7-56 U/L	↑ Elevated
ALP	140 U/L	44-147 U/L	Mildly elevated
CD4+ T-cell count	1 cell/µL	500-1,500 cells/µL	↓ Profound immunosuppression

Because no clear source of infection was identified, a lumbar puncture and an extensive infectious workup were performed while blood cultures obtained in the emergency department remained pending. Results were negative for the following: respiratory viral panel, including influenza A and B, respiratory syncytial virus, SARS-CoV-2, parainfluenza virus types 1-4, human metapneumovirus, adenovirus, and rhinovirus/enterovirus; fungal blood culture; acid-fast bacilli (AFB) culture; cerebrospinal fluid (CSF) culture; CSF fungal culture; CSF AFB culture; John Cunningham (JC) virus DNA by polymerase chain reaction (PCR); rapid plasma reagin (RPR); CSF Venereal Disease Research Laboratory (VDRL) test; serum and CSF cryptococcal antigen; serum and urine histoplasma antigen and antibody; Fungitell β-D-glucan assay; parvovirus B19 immunoglobulin G (IgG) and immunoglobulin M (IgM); serum and CSF Toxoplasma IgG and IgM; QuantiFERON-TB Gold Plus; and the CSF meningoencephalitis panel, which included cytomegalovirus, enterovirus, herpes simplex virus type 1, herpes simplex virus type 2, human herpesvirus 6, human parechovirus, varicella-zoster virus, *Escherichia coli* K1, *Haemophilus influenzae*, *Listeria monocytogenes*, *Neisseria meningitidis*, *Streptococcus agalactiae*, *Streptococcus pneumoniae*, and *Cryptococcus neoformans*/*Cryptococcus gattii*. Epstein-Barr virus (EBV) nuclear antigen IgG was reactive, consistent with prior exposure, whereas EBV IgM was nonreactive, indicating no evidence of acute infection (Table 2).

**Table 2:** Negative infectious workup results AFB: acid-fast bacilli, CSF: cerebrospinal fluid, JC: John Cunningham, DNA: deoxyribonucleic acid, PCR: polymerase chain reaction, RPR: rapid plasma reagin, VDRL: Venereal Disease Research Laboratory, IgG: immunoglobulin G, IgM: immunoglobulin M, TB: tuberculosis, EBV: Epstein-Barr virus

Test	Specimen source	Result
Respiratory viral panel	Nasopharyngeal swab	No virus detected
Fungal culture	Blood	No growth
AFB culture	Blood	No growth
CSF culture	CSF	No growth
CSF fungal culture	CSF	No growth
CSF AFB culture	CSF	No growth
JC virus DNA (PCR)	CSF	Not detected
RPR	Serum	Non-reactive
CSF VDRL	CSF	Non-reactive
Cryptococcus antigen	Serum and CSF	Negative
Histoplasma antigen	Urine	Negative
Histoplasma antigen	Serum	Negative
Fungitell β-D-glucan	Serum	Negative
Parvovirus B19 IgG	Serum	Negative
Parvovirus B19 IgM	Serum	Negative
Toxoplasma IgG	Serum and CSF	Negative
Toxoplasma IgM	Serum and CSF	Negative
Meningoencephalitis panel	CSF	No organisms detected
QuantiFERON-TB Gold Plus	Blood	Negative
EBV IgM antibody	Serum	Non-reactive (no acute infection)

**Figure 1:**
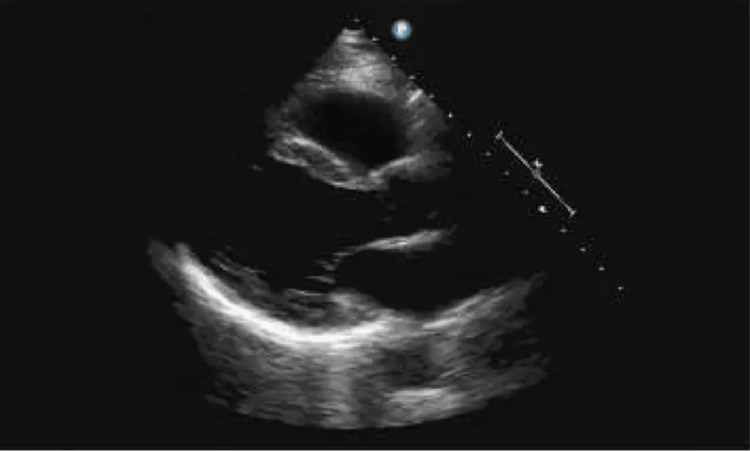
TTE (parasternal long-axis view) demonstrating the left ventricle and mitral valve without evidence of vegetations TTE: transthoracic echocardiogram

Ultimately, two sets of blood cultures became positive for gram-positive bacilli on hospital day 1 and were later identified as *Brevibacterium* spp. A transthoracic echocardiogram (TTE) demonstrated no valvular abnormalities or vegetations, with a preserved left ventricular ejection fraction of 65% (reference range: 55-70%) (Figure 1). A transesophageal echocardiogram was not pursued because the TTE provided adequate image quality, showed no evidence of valvular abnormalities or vegetations, and the blood cultures cleared promptly with antibiotic therapy. Notably, the patient's CD4+ T-cell count was 1 cell/µL (reference range: 500-1,500 cells/µL).

As part of the evaluation for the falls that led to her admission, the patient underwent assessment for an acute neurologic process. No focal neurologic deficits were identified on examination, and neuroimaging showed no acute intracranial pathology. Cardiogenic causes of syncope were excluded based on normal sinus rhythm on electrocardiography, an unremarkable echocardiogram, and the absence of arrhythmic events on continuous telemetry monitoring.

The patient was treated with intravenous vancomycin for *Brevibacterium* bacteremia. Antimicrobial susceptibility testing demonstrated susceptibility to vancomycin, supporting the treatment regimen. Given her profound immunosuppression and the presence of oral thrush, candidal esophagitis was suspected. However, because she denied dysphagia and odynophagia, upper endoscopy was deferred, and empiric micafungin therapy was initiated. She also received trimethoprim-sulfamethoxazole for primary prophylaxis against *Pneumocystis jirovecii* pneumonia. Prophylaxis against *Mycobacterium avium* complex was not indicated because she had been restarted on ART following her previous nonadherence. A two-week course of intravenous vancomycin was completed, with treatment duration guided by clearance of blood cultures. Repeat blood cultures confirmed resolution of the bacteremia before antimicrobial therapy was discontinued. The patient was discharged with instructions to follow up with gastroenterology for a colonoscopy to evaluate whether the gastrointestinal tract was the source of infection.

## Discussion

*Brevibacterium* spp., historically regarded as nonpathogenic skin commensals, have emerged in recent years as opportunistic pathogens, primarily affecting immunocompromised individuals, particularly those with indwelling devices [3-5]. These aerobic, catalase-positive, non-motile, Gram-positive rods are frequently misidentified morphologically as diphtheroids on Gram stain, which can delay accurate diagnosis [1]. Although reported cases remain limited, an increasing body of evidence suggests that *Brevibacterium* spp. can cause clinically significant infections, including catheter-related bloodstream infections, peritonitis, endocarditis, and central nervous system infections [6,7]. The introduction of matrix-assisted laser desorption ionization-time-of-flight mass spectrometry has substantially improved species-level identification of rare organisms such as *Brevibacterium*, which may partly explain the increasing number of reported cases [8].

In a narrative review by Panayiotou et al., 41 studies involving 42 patients with *Brevibacterium* spp. infections were analyzed [8]. Bloodstream infection was the most common clinical presentation (57.1%), followed by peritoneal dialysis-associated peritonitis (16.7%). The principal predisposing factors were the presence of central venous catheters, malignancy, and end-stage renal disease, particularly among patients undergoing peritoneal dialysis [8]. However, our case demonstrates that *Brevibacterium* can cause invasive infection even in the absence of traditional device-related risk factors, such as an indwelling central venous catheter or prosthetic device, highlighting that severe immunosuppression alone may be sufficient to predispose patients to invasive *Brevibacterium* disease.

Available antimicrobial susceptibility data indicate that *Brevibacterium* spp. frequently exhibit resistance or intermediate susceptibility to β-lactam antibiotics, particularly penicillin and ceftriaxone [2,3]. In contrast, susceptibility to vancomycin remains consistently high, making it the preferred empiric therapy while culture and antimicrobial susceptibility results are pending [2]. Aminoglycosides have also been used successfully as part of combination therapy [7].

## Conclusions

*Brevibacterium* spp. are part of the normal human skin microbiota and are often presumed to be the source of infection in immunocompromised patients. Although profound immunosuppression is a well-recognized risk factor for *Brevibacterium* bacteremia, the most commonly reported predisposing factors include the presence of central venous catheters, malignancy, and end-stage renal disease. Notably, our patient had neither an indwelling central venous catheter nor a prosthetic device, underscoring that device-related risk factors are not essential for the development of invasive *Brevibacterium* infection in the setting of severe immunosuppression. This case highlights that *Brevibacterium* bacteremia may arise from alternative sources in profoundly immunocompromised patients and emphasizes that timely recognition and vancomycin treatment can yield favorable outcomes.
